# Supplementary material for: Higher Bifidobacterium spp. fecal abundance is associated with a lower prevalence of hyperglycemia and cardiovascular risk markers among schoolchildren from Bahia, Brazil
Source: PLoS One. 2023 Oct 19;18(10):e0290813. doi: 10.1371/journal.pone.0290813 (PMC10586616; doi:10.1371/journal.pone.0290813)
Supplement: Attachment — Submitted filename: pone D review 1.6.2023.pdf [file pone.0290813.s004.pdf]

Reviewer Attachments for Manuscript Number PONE-D-22-33730 "Higher Bifidobacterium spp. fecal abundance is associated with a lower prevalence of hyperglycemia and cardiovascular risk markers among schoolchildren from Bahia, Brazil"

1	The work is indeed a good piece of work and shows the relevance in current context with regard to food intake and gut flora behaviors string response in research field
2	The early reviewers (both) have addressed major questions and the author also tried with its maximum effort to address the queries with responsibility
3	I have only to say about the discussion part where statistical interpretation if possible can be addressed
4.	Rest I believe the manuscript is good to consider for publication

Name: Dr.Mousumi Das

Date : 01.06.2023
