# Supplementary material for: Higher Bifidobacterium spp. fecal abundance is associated with a lower prevalence of hyperglycemia and cardiovascular risk markers among schoolchildren from Bahia, Brazil
Source: PLoS One. 2023 Oct 19;18(10):e0290813. doi: 10.1371/journal.pone.0290813 (PMC10586616; doi:10.1371/journal.pone.0290813)
Supplement: Attachment — Submitted filename: Response to the Reviewers.docx [file pone.0290813.s001.docx]

Response to Academic Editor and Reviewers

Dear Academic Editor and Reviewers,

We are thankful for the time and attention dedicated to this first review of our manuscript “Higher *Bifidobacterium* spp. fecal abundance is associated with a lower prevalence of hyperglycemia and cardiovascular risk markers among schoolchildren from Bahia, Brazil.” Each point raised by the review is explored as follows.

**Editor comments:**

**Point 1:** The work brings important considerations about the nutritional conditions of Brazilian children, presenting a promising health marker. However, it lacks English revision, and that the tables are clearer, presenting results from only one type of statistical analysis.

**Response 1:** Thank you for this positive feedback. The manuscript underwent another English revision. Regarding the tables, unfortunately, it is not possible to standardize the statistical analysis because some of the numerical variables have two groups and others have three or more groups, which leads to apply different statistical tests to compare them.

**Journal Requirements:**

**Point 1:** Please ensure that your manuscript meets PLOS ONE's style requirements, including those for file naming.

**Response 1:** We double-checked the style requirements and hope that the revised manuscript submitted achieves them.

**Point 2:** We note that the grant information you provided in the ‘Funding Information’ and ‘Financial Disclosure’ sections do not match.

**Response 2:** During the manuscript’s writing, the first author received a research grant from the Foundation for Research Support of the State of Bahia – Fundação de Amparo à Pesquisa do Estado da Bahia (FAPESB, Brazil). We intend to standardize this information during the resubmission process.

**Point 3:** In your Data Availability statement, you have not specified where the minimal data set underlying the results described in your manuscript can be found. PLOS defines a study's minimal data set as the underlying data used to reach the conclusions drawn in the manuscript and any additional data required to replicate the reported study findings in their entirety. All PLOS journals require that the minimal data set be made fully available.

**Response 3:** The dataset used to support the results presented in the manuscript is in process of upload at the FIOCRUZ institutional data repository (<https://dadosdepesquisa.fiocruz.br/>; ARCA DADOS). A specific DOI number will be attributed to the dataset and will be informed prior to the publication.

**Point 4:** PLOS requires an ORCID iD for the corresponding author in Editorial Manager on papers submitted after December 6th, 2016. Please ensure that you have an ORCID iD and that it is validated in Editorial Manager. To do this, go to ‘Update my Information’ (in the upper left-hand corner of the main menu), and click on the Fetch/Validate link next to the ORCID field. This will take you to the ORCID site and allow you to create a new iD or authenticate a pre-existing iD in Editorial Manager.

**Response 4:** The corresponding author has an Editorial Manager validated ORCID iD and the information was updated.

**Point 5:** While revising your submission, please upload your figure files to the Preflight Analysis and Conversion Engine (PACE) digital diagnostic tool, <https://pacev2.apexcovantage.com/>.

**Response 5:** We used the PACE digital diagnostic tool, and the figures now attend to the PLOS One requirements.

We hope these changes attend to the points raised by the first Academic Editor review.

Best regards,

**Ricardo Riccio Oliveira**

Instituto Gonçalo Moniz, Fundação Oswaldo Cruz (Fiocruz)

+55 71 3176-2347 | +55 71 3176-2266

ricardo.riccio@fiocruz.br
