# Supplementary material for: Higher Bifidobacterium spp. fecal abundance is associated with a lower prevalence of hyperglycemia and cardiovascular risk markers among schoolchildren from Bahia, Brazil
Source: PLoS One. 2023 Oct 19;18(10):e0290813. doi: 10.1371/journal.pone.0290813 (PMC10586616; doi:10.1371/journal.pone.0290813)
Supplement: Attachment — Submitted filename: Bifidobacteria March 2023.docx [file pone.0290813.s002.docx]

This is a very interesting manuscript.

I have a few questions or suggestions:

1. The first paragraph in Discussion would probably fit better to Introduction (incorporated to the current Introduction).
2. In the paragraph starting on line 320: any previous information in literature how diet would modify the *Bifidobacterium* ssp. in the gut microbiota after breastfeeding stopped?
3. Who carried out the 24-hr recall? Were they trained to carry out the recall (that can be quite challenging if the interviewer does not know for example the local food supply well, and/or not mastering the serving size estimation and conversion to weight units)? How long did children fast before the blood draw?
4. How was the food consumption information from 24-hr recall forms and food composition tables converted into data on dietary intake (energy and macronutrients) of a child/teenager: did you use a specific in-house program or an application for it? One-time 24-hr recall is not ideal in studying dietary intake on individual level because diet can vary a lot from day to day. Not very strong conclusions can be made based on dietary intake (one-time 24-hr recall).
5. There were several dietary biomarkers measured from the blood samples like ferritin, vitamins B12 and D. The results are missing. If you don’t want to report them please do not mention them in the methods.
6. Any adjustments in the statistical analyses? Age could be a confounder.
7. Table 1. You indicate that 179 children were breastfed at infancy. However, if you sum up the N’s in two categories of BF durations (108+79) you get 187 children. Where is the difference coming from?
8. It would be clearer if you first give the number of children breastfed <6 months (n=79) and then those breastfed ≥6 months (n=108). I think “≥6 months” is clearer than “Up to sixth month” and “<6 months” instead of “Less than sixth month”. This is a matter of taste of course.
9. It says in line 196 “exclusive breastfeeding” but should probably be any breastfeeding or just ‘breastfeeding’ to match with Table 1. Hard to believe that 58% of the children were exclusively breastfed by age of 6 months or longer (no formula or solid foods). This is probably referring to any breastfeeding.
10. In Table 2 I would say “Energy (kcal)”.
11. Please use Fats instead of Lipids.
12. In the foot note or somewhere else there should be a brief definition of “processed foods” and of “ultra-processed foods” so that the reader understands what is the difference between these two types of foods.
13. Please check the use of comma and period in the text. Now it is not consistent.
14. Studying the topic further including physical activity sounds very good! All the best for it!
