# Supplementary material for: Higher Bifidobacterium spp. fecal abundance is associated with a lower prevalence of hyperglycemia and cardiovascular risk markers among schoolchildren from Bahia, Brazil
Source: PLoS One. 2023 Oct 19;18(10):e0290813. doi: 10.1371/journal.pone.0290813 (PMC10586616; doi:10.1371/journal.pone.0290813)
Supplement: Attachment — Submitted filename: Response to Reviewers.docx [file pone.0290813.s005.docx]

Response to Reviewers

Dear reviewers,

We are thankful for the time and attention dedicated to this third review of our manuscript “Higher *Bifidobacterium* spp. fecal abundance is associated with a lower prevalence of hyperglycemia and cardiovascular risk markers among schoolchildren from Bahia, Brazil.” We fully understand that all these contributions are important for the quality of the final work. The points raised by the reviewers are explored as follows.

**Reviewer 3**

**Comment 1:** The work is indeed a good piece of work and shows the relevance in current context with regard to food intake and gut flora behaviors string response in research field.

**Response:** We are grateful for your positive feedback on our work. It's encouraging to hear that you find the relevance of our study in the current context of food intake and its impact on the gut microbiome. We will continue to strive for excellence in our research endeavors.

**Comment 2:** The early reviewers (both) have addressed major questions and the author also tried with its maximum effort to address the queries with responsibility.

**Response:** Thank you for recognizing our commitment to addressing the reviewers' concerns. We have indeed put forth our utmost effort to respond to the queries responsibly. Your acknowledgment is truly motivating as we aim to ensure the quality and rigor of our work.

**Comment 3:** I have only to say about the discussion part where statistical interpretation if possible can be addressed.

**Response:** We appreciate your suggestion regarding the discussion section and the inclusion of a statistical interpretation. We have considered your input and worked towards enhancing the depth of analysis in that part of the paper and included a sentence in the last paragraph of the section.

**Comment 4:** Rest I believe the manuscript is good to consider for publication.

**Response:** Thank you for considering our manuscript favorably. Your confidence in the quality of our work is truly motivating. We will ensure that any remaining aspects are polished to meet the standards for publication.

**Reviewer 4**

**Comment 1:** The authors performed a cross-sectional analysis of the relationship between the fecal abundance of *Bifidobacterium* spp. with dietary and anthropometric parameters among the school-aged population. The analyses linked positive metabolic outcomes with increased *Bifidobacterium* spp. fecal abundance. The data provide a framework to further understand the effects of *Bifidobacterium* spp. on children's metabolic outcomes. The authors were responsive to reviewers' suggestions; most revisions and select omissions are justified. However, there are still some concerns that need to be fully addressed.

**Response:** We sincerely appreciate your thorough review of our manuscript and your acknowledgment of our efforts to address previous reviewers' suggestions. It's encouraging to know that most of the revisions and omissions were justified and well-received. We value your feedback on the remaining concerns, and we are committed to fully addressing them to ensure the overall quality of the manuscript.

**Comment 2:** One major missing element is the current state of knowledge about the field. The authors should accurately represent appropriate studies and references in the Introduction section.

**Response:** Your observation about the need to include a comprehensive overview of the current state of knowledge in the field is invaluable. We have diligently worked on enhancing the Introduction section by accurately representing relevant studies and incorporating appropriate references. Your guidance will undoubtedly contribute to strengthening the overall context and significance of our work.

**Point 1:** The current version is not cohesive and lacks sufficient background, including but not limited to: - Line 73-75: missing valuable reference for the statement about “conflicting results”. The previous studies about the relationship between *Bifidobacterium* spp. and metabolic outcomes in the child population should be referenced for context.

**Response:** We acknowledge the need for better cohesion and background in the current version. We addressed the missing reference and included relevant studies on the relationship between *Bifidobacterium* spp. and metabolic outcomes, including in children, to provide the necessary context (references 16 to 19 - Michels N. et al, 2022; Gholizadeh P. et al, 2019; Guo Q. et al, 2019; Chen AC. et al, 2022).

**Point 2:** Line 70: The authors should double-check the number. According to reference 3 and current NCBI Taxonomy ID 1678, there are more than 29 *Bifidobacterium* species.

**Response:** Thank you for bringing this to our attention. We have carefully reviewed the reference (Hidalgo-Cantabrana C. et al, 2017) and found that there are indeed more than 50 documented species of *Bifidobacterium*. We have updated our information accordingly in the first paragraph of page 3.

**Point 3:** Line 62: The evidence of “tropism” cannot be found in reference 2. Based on reference 2, one of the significant functions of gut microbiota is controlling epithelial cell proliferation and differentiation, but “tropism” was not mentioned in reference 2.

**Response:** The text mentions that the trophic function of the intestinal microbiota occurs through the stimulation of proliferation and differentiation of the intestinal epithelium, as well as the development and modulation of the immune system. This information was described in previous reference 1 (Beserra BTS, 2014 – the first paragraph of page 31) and not in the previous reference 2 (Gomaa EZ, 2020). Nevertheless, the suggestion to provide new references to better contextualize the topic has been addressed, and we have included the following: Manos J, 2022; Liang X. et al, 2022; Krakovski MA. et al, 2022; Zhang W. et al, 2022; Aleman RS, Moncada M, Aryana KJ, 2023; Dowling LR. et al, 2022 (references 6 to 11).

We double-checked the reference list to ensure its completeness and alignment with PLOS One specifications. We hope these changes attend to the comments and points raised by the reviewers.

Best regards,

**Ricardo Riccio Oliveira**

Instituto Gonçalo Moniz, Fundação Oswaldo Cruz (Fiocruz)

+55 71 3176-2347 | +55 71 3176-2266

ricardo.riccio@fiocruz.br
